# Network Analysis of Epidermal Growth Factor Signaling Using Integrated Genomic, Proteomic and Phosphorylation Data

**DOI:** 10.1371/journal.pone.0034515

**Published:** 2012-03-29

**Authors:** Katrina M. Waters, Tao Liu, Ryan D. Quesenberry, Alan R. Willse, Somnath Bandyopadhyay, Loel E. Kathmann, Thomas J. Weber, Richard D. Smith, H. Steven Wiley, Brian D. Thrall

**Affiliations:** 1 Computational Biology and Bioinformatics, Pacific Northwest National Laboratory, Richland, Washington, United States of America; 2 Biological Separations and Mass Spectrometry, Pacific Northwest National Laboratory, Richland, Washington, United States of America; 3 Cell Biology and Biochemistry, Pacific Northwest National Laboratory, Richland, Washington, United States of America; 4 Statistical Sciences, Pacific Northwest National Laboratory, Richland, Washington, United States of America; 5 Environmental Molecular Sciences Laboratory, Pacific Northwest National Laboratory, Richland, Washington, United States of America; Rikagaku Kenkyūsho Center for Allergy and Immunology, Japan

## Abstract

To understand how integration of multiple data types can help decipher cellular responses at the systems level, we analyzed the mitogenic response of human mammary epithelial cells to epidermal growth factor (EGF) using whole genome microarrays, mass spectrometry-based proteomics and large-scale western blots with over 1000 antibodies. A time course analysis revealed significant differences in the expression of 3172 genes and 596 proteins, including protein phosphorylation changes measured by western blot. Integration of these disparate data types showed that each contributed qualitatively different components to the observed cell response to EGF and that varying degrees of concordance in gene expression and protein abundance measurements could be linked to specific biological processes. Networks inferred from individual data types were relatively limited, whereas networks derived from the integrated data recapitulated the known major cellular responses to EGF and exhibited more highly connected signaling nodes than networks derived from any individual dataset. While cell cycle regulatory pathways were altered as anticipated, we found the most robust response to mitogenic concentrations of EGF was induction of matrix metalloprotease cascades, highlighting the importance of the EGFR system as a regulator of the extracellular environment. These results demonstrate the value of integrating multiple levels of biological information to more accurately reconstruct networks of cellular response.

## Introduction

Systems biology is an approach to develop comprehensive and ultimately predictive models of how components of a biological system give rise to its observed behavior [1], [2]. Because of the complexity of biological organisms, however, this approach has proven most successful when applied to relatively small-scale systems [3]. Applications to more significant and complex problems have recently been enabled by technical advances in molecular biology and genome sequencing, which generate high-dimensional data with the appropriate throughput and sensitivity. Genome-wide mRNA expression profiling using cDNA and oligonucleotide microarrays or serial analysis of gene expression have proven valuable in identifying mRNA expression changes associated with disease, metabolic states, development and exposure to drugs and environmental agents [4], [5], [6], [7]. More recent advances in mass spectrometry (MS)-based proteomics using stable isotope labeling have made quantitative protein profiling, including measures of post-translational protein modification, feasible at a global scale [8], [9], [10]. A variety of other technologies capable of providing high-dimensional biological response data has also emerged, including multiplexed protein microarrays, flow cytometry, and two-hybrid systems for mapping protein interactions [11], [12], [13], [14]. Datasets derived from these technologies can potentially provide a foundation for building quantitative models of biological systems but only if they can be integrated into a coherent relational network of cellular response.

Most current high-throughput technologies only provide data for a single molecule type, and the underlying regulatory structure of the cell must be inferred from their qualitative or quantitative relationships. Data describing only a single level of biological regulation is unlikely to fully explain the behavior of complex biological systems. Thus, there is a need for integrating data from multiple sources representing different hierarchical levels of regulation to reconstruct more complete cellular networks. For example, studies comparing mRNA and protein expression profiles have indicated that mRNA changes are unreliable predictors of protein abundance [15], [16]. Mathematical modeling of these processes suggests that understanding the regulation of simple cellular networks requires data describing the dynamics of both mRNA and protein expression levels [17]. Estimating steady-state mRNA and protein changes from a single time point, however, can be misleading because of the time needed for protein synthesis and degradation. To our knowledge, temporal-based analyses of correlations between global protein and gene expression patterns in human cells have yet to be reported.

The necessity for integrated data analysis across ‘omics platforms is further driven by the desire to identify fundamental properties of biological networks, such as redundancy, modularity, robustness, and feedback control [1], [18], [19]. Such properties provide the underlying structure of signaling networks, yet they are difficult to specify using a single type of analytical measurement. While the need for data integration is clearly recognized, in practice there are few reported examples that quantify the benefits gained by this approach, particularly for mammalian systems. Notably, little effort has been made to systematically evaluate the degree of information overlap provided by different types of ‘omics data and how they can distinctly inform network and pathway analyses. This is despite the fact that all high-throughput technologies have varying sampling efficiencies and systematic biases and limitations that give rise to different false positive and false negative rates. Consequently, it is unclear whether the cell response pathways revealed by integrating microarray and proteomic data will be more informative than those inferred by global mRNA microarray data alone.

To explore the practicality of integrating different types of high-throughput data to understand complex cellular functions, we have conducted a multidimensional analysis of the temporal response of human mammary epithelial cells (HMEC) to epidermal growth factor receptor (EGFR) activation. EGFR signaling plays an important role in regulating proliferation and motility in many epithelial cells and can integrate signals from diverse pathways through receptor crosstalk [20]. Both the proliferation and motility of HMEC require EGFR activation [21], [22]. Conveniently, HMEC can be arrested in the cell cycle by removing EGF in the culture medium, and subsequently induced to synchronously reenter the cell cycle by re-addition of EGF. This allows examination of the dynamics of the mitogenic response without using inhibitors or other non-physiological treatments.

In this study, we examined time-dependent changes in gene and protein expression patterns following EGF treatment of synchronized HMEC to determine how data from each platform qualitatively contributed to pathway and network analysis. While the cell processes identified by each data type varied significantly, integration of the multiple datasets recapitulated most of the known mitogenic pathways mediated through EGFR. Furthermore, networks derived from the combined datasets exhibited more highly connected signaling nodes with an appropriate hierarchical structure than networks derived from any individual dataset. This study demonstrates that integration of multiple data types provides complementary, not redundant, information necessary to reconstruct complex cellular response networks.

## Results

### Changes in Gene and Protein Expression during the HMEC Mitogenic Response

**Figure 1:**
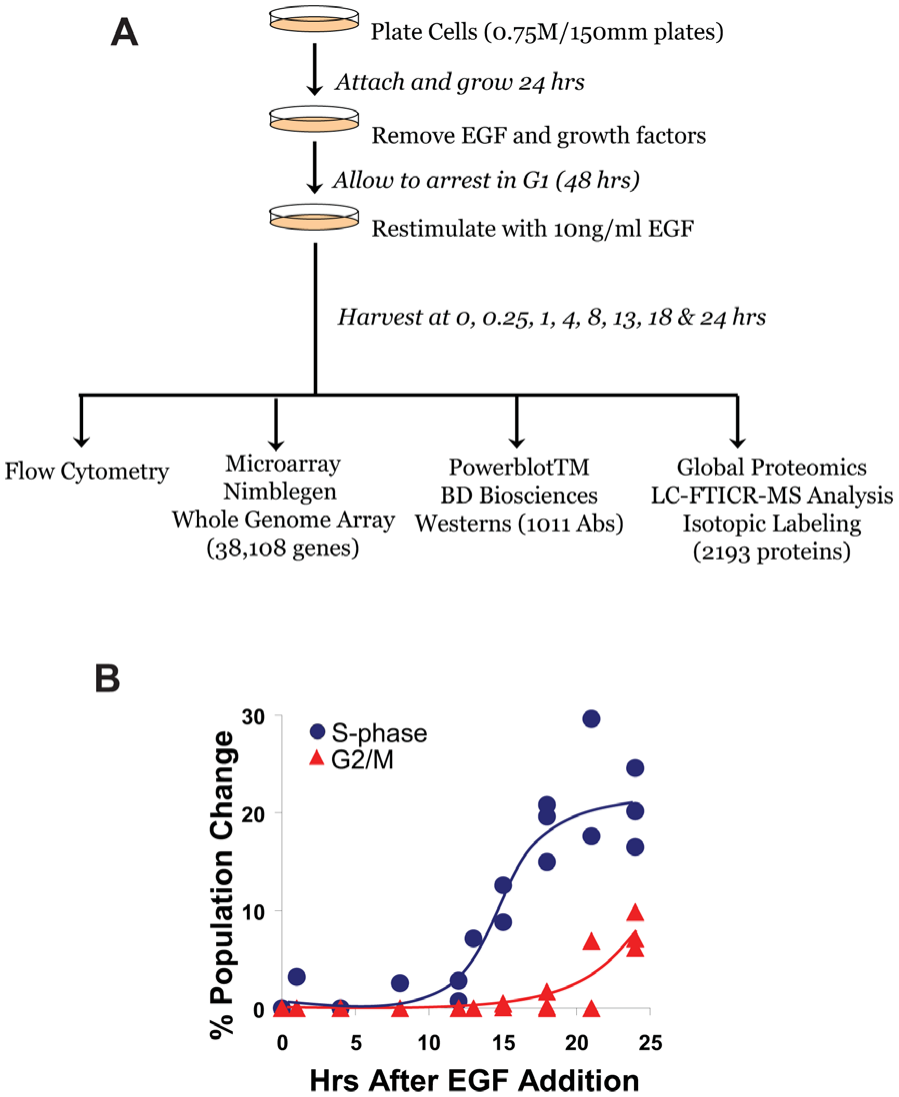
Experimental design and characterization of cell cycle transition with EGF treatment. A) Experiments were scaled to provide sufficient sample for parallel analyses by gene microarray, global proteomics and Western blot technologies. B) Flow cytometry results showing the time course for transitions between G_1_/S and G_2_/M phases during EGF-induced mitosis.

To determine the degree to which changes in common cell regulatory networks are detected by different high-content data technologies, we analyzed the temporal response of cells to activation of EGFR using whole genome microarrays, high throughput Western blots (Powerblot™), and mass spectrometry-based proteomics. The experimental strategy (Fig. 1A) used takes advantage of the inherent dependence of HMEC on EGFR autocrine signaling for normal cell proliferation. Previous studies have shown that removal of EGFR ligands from culture medium or blocking EGFR activation causes HMEC to reversibly arrest in G_0_/G_1_ of the cell cycle [22]. We used this approach to induce a synchronized EGFR signaling response and facilitate network analysis from time-course data. At different times following EGF addition, we collected sufficient sample for parallel microarray, Western blot, and MS-based proteomics analysis (Fig. 1A). Monitoring the G_1_-S-G_2_/M transitions by flow cytometry (Fig. 1B) also yielded a temporal benchmark for the EGF-induced signaling responses. Removal of serum and growth factors caused >95% of the cells to arrest in G_0_/G_1_ phase. Subsequent addition of EGF induced a G_1_-S transition between 13–14 hr, followed by entry into G_2_/M between 18–22 hr (Fig. 1B).

**Figure 2:**
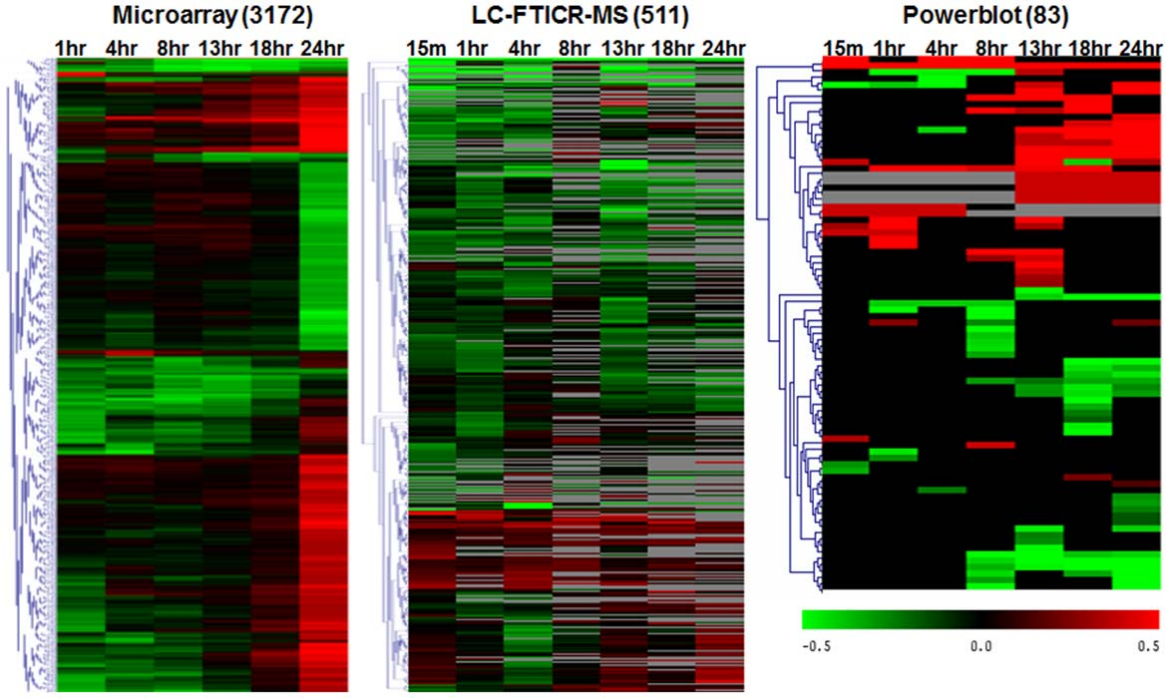
Hierarchical cluster analyses showing temporal changes in expression ratios for significant RNA and protein changes. The scale bar indicates the log_10_ expression ratio compared to 0 hr controls. Values in gray indicate the protein/phosphorylated protein was not detected at that time point.

A complete listing of the significant RNA and protein changes identified by each platform is provided in the supplementary information (Table S1). Among 38,108 probesets on the microarray platform, 3172 RNA expression profiles were found to significantly vary from the control (0 hr) based on analysis of variance, false discovery rate (FDR) calculation (5%) and a 1.5-fold change threshold (relative to control) in at least one of the time points (Fig. 2). Since our overall goal was to evaluate the impact of data integration in assessing global cell responses, these criteria were set to maximize the overlap of potential gene changes between datasets.

Categorically, RNAs that changed represented a wide variety of cell processes including immediate early genes, cell cycle regulatory genes, anti-apoptotic genes, matrix remodeling and proteolytic genes. The most highly-induced genes identified across all time points examined were extracellular proteases, including interstitial collagenase (MMP1, 24-fold induction), and stromelysin 2 (MMP10, 8-fold induction). We also observed early gene induction patterns, including activating transcription factor 3 (ATF3) and prostaglandin-endoperoxide synthase 2 (PTGS2), which were transiently up-regulated at 1 hr and returned to basal levels by 4 hr. A dramatic change in gene expression at the 24 hr time point was observed, reflecting the shift in cell population to G_2_/M measured by flow cytometry.

To measure changes in protein level by MS, we used the accurate mass and time (AMT) tag strategy [23]. In this approach, peptides (AMT tags) were identified using liquid chromatography-Fourier transform ion cyclotron resonance mass spectrometry (LC-FTICR) analysis, based on both accurate mass measurements and chromatographic elution times in reference to a mass tag database of proteins expressed in HMEC [9], [24], [25], [26]. The advantages of this approach have been previously described [23] and include increased sample throughput and dynamic range compared to typical LC-MS/MS analyses, as well as providing a measure of protein abundance. Using conservative criteria previously described [9], 2193 proteins were identified in the EGF-treated HMEC samples by LC-FTICR. A stable isotope labeling approach that employs quantitative exchange of either ^16^O or ^18^O at the site of trypsin digestion was also used to estimate protein abundances [9]. This approach permitted ratiometric analysis of the LC-FTICR results similar to that commonly used for RNA microarray data. Based on the calculated ion intensity ratios of treated to control (^18^O/^16^O), and using a significance threshold of 1.5-fold change, 511 proteins (with ≥2 peptide identifications each) were found to change in abundance in response to EGFR activation (Fig. 2). The 1.5-fold significance level was chosen to maximize potential overlap between the different datasets based on our previous analyses that demonstrate a high reproducibility for the ^18^O/^16^O labeling method [9] and is consistent with analogous global proteomic studies [27].

The antibody-based proteomic analysis by Western blots from triplicate analyses indicated that 83 out of 612 detected proteins showed a ≥1.5 fold difference in abundance in response to EGF treatment (at any time point) compared to 0 hr controls (Fig. 2). These analyses also included antibodies that selectively recognize phosphorylated proteins, providing an additional dimension of data. Among the total proteins identified by Western analysis, 20 were phosphorylated, and 9 of these significantly changed phosphorylation levels after adding EGF. These included rapid increases in phosphorylation of SRC, MAPK1 (ERK2), and STAT3 consistent with the ability of EGFR stimulation to induce phosphorylation of these signaling molecules [10]. The temporal pattern of ERK2 phosphorylation during the HMEC mitogenic response was biphasic, similar to results from our previous studies of ERK2 activation patterns regulated by EGFR transactivation [20]. Additional proteins that were phosphorylated in response to EGFR activation included catenin delta 1(CTNND1), mitogen-activated protein kinase 14 (MAPK14), and c-AMP-dependent protein kinase type 2 (PRKAR2B).

### Data Integration

**Figure 3:**
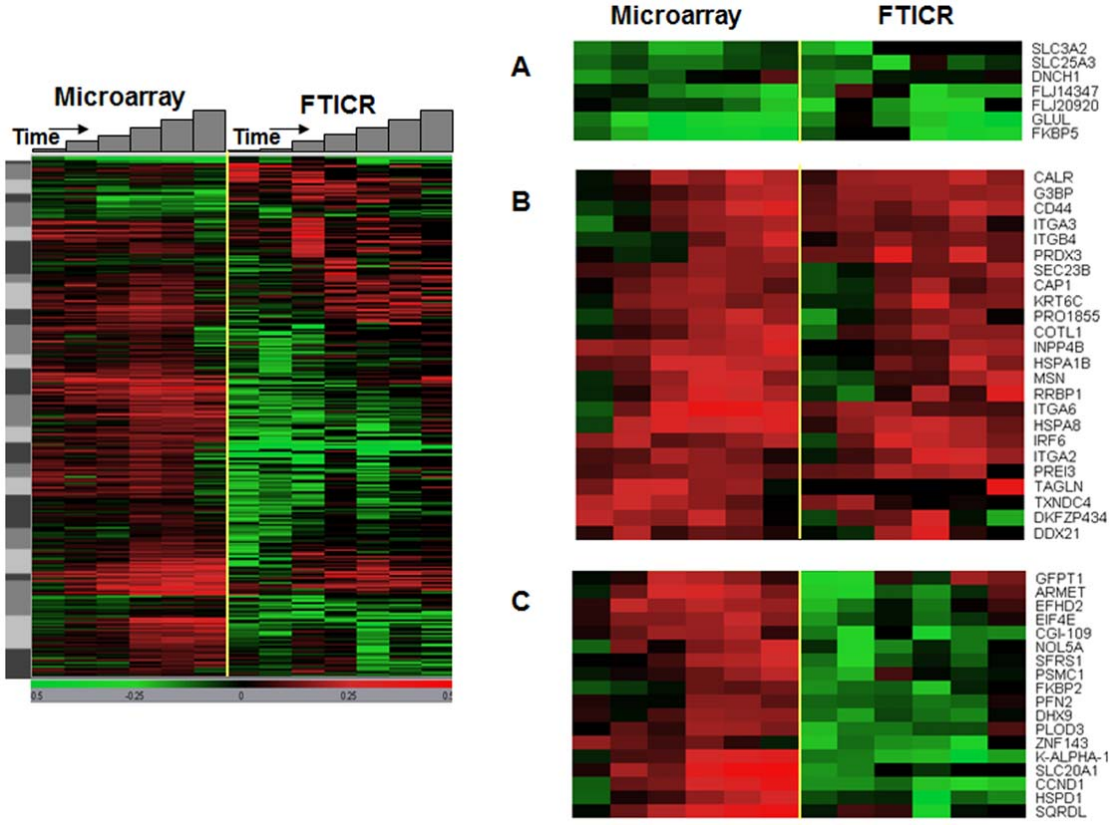
K-means cluster analysis comparison of microarray and LC-FTICR expression ratio data. The left panel shows the overall results for 446 RNA/protein pairs expressed as the log_10_ ratio over 0 hr control samples. Panels A–C highlight 3 different clusters that show different overall temporal patterns between the RNA and protein data.

To estimate the degree of concordance between RNA and protein expression changes, the subset of proteins observed to change by LC-FTICR analysis (511) was merged with the corresponding gene expression values measured on the microarray platform. By cross-referencing platform-specific identifiers to common gene symbols [28], 446 RNA/protein pairs could be reliably cross-indexed and analyzed. We first used k-means cluster analysis (Fig. 3) to assess the heterogeneity between measured RNA and protein expression profiles, which may represent differential mechanisms of regulation. Some clusters of RNA/protein pairs show a classical pattern of regulation, where RNA changes preceded or coincided with a corresponding change in protein abundance (Fig. 3, clusters A,B). Other clusters show complex patterns that imply feedback processes between protein half-life and compensatory RNA induction. For example, the relative abundance of proteins in cluster C generally decreased at early times following EGFR activation (Fig. 3, cluster C). The decline in protein abundance for this cluster was followed by an apparent increase in RNA synthesis, which was then followed by an increase in protein abundance to higher levels by 24 hr. In other clusters, the concordance between RNA and protein expression changes was not apparent, suggesting that steady-state changes in RNA and protein abundance in those species were regulated independently.

**Figure 4:**
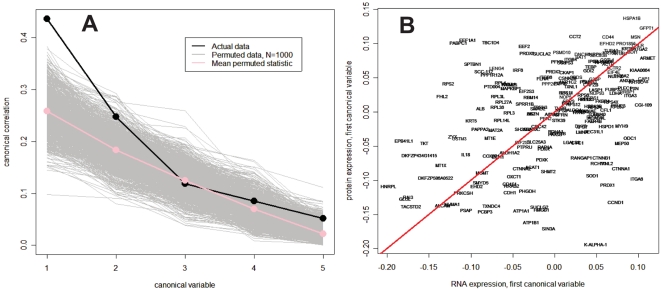
Canonical correlation analysis of RNA and protein temporal expression profiles. A) Ranked canonical correlations between RNA expression and protein expression, compared with random permutations. Each gray line shows the computed canonical correlations for a single random permutation. The first observed canonical correlation exceeds all corresponding permuted values, and the second canonical correlation exceeds 99% of permuted values; both are considered statistically significant. B) Scatter plot of first canonical variable between RNA and protein expression for 199 gene/protein pairs with a correlation coefficient of 0.44.

Because multiple time points were measured in this study, multivariate correlation analysis was also used to assess the global correlation between RNA expression and protein abundance changes over time. Canonical correlation analysis was used to identify a linear combination of time points that maximizes the correlation between RNA and protein expression profiles [29]. Since RNA and protein expression may proceed at different rates, this approach facilitated identifying multiple, non-obvious correlations between the two expression profiles. For this analysis, only RNA/protein pairs with complete data across all time points were used, resulting in 199 pairs. We evaluated the significance of the observed canonical correlations by randomizing the protein identifiers while maintaining the data for individual time profiles intact and canonical correlations for each of 1000 permutations were calculated. The first observed canonical correlation exceeds all corresponding permuted (randomized) values, and the second canonical correlation exceeds 99% of permuted values, so both are considered statistically significant (Fig. 4A). A scatter plot of first canonical variable between RNA and protein expression for the 199 genes shows a correlation coefficient of 0.44 (Fig. 4B). This result is generally consistent with previous studies that have examined correlations between gene and protein abundance at single time points [27], [30], [31], [32]. These data imply that even when time course data is compared, a significant fraction of the protein abundance changes were not tightly coupled with a corresponding temporal change in RNA expression, providing strong evidence for substantial post-transcriptional regulation of protein expression following growth factor stimulation.

The overall smoothed shapes of the temporal RNA and protein expression patterns derived by regression were also compared and ranked using a test F-statistic in order to identify some of the most concordant and discordant RNA/protein pairs (Figure S1). An important question is whether there is any relationship between the degree of concordance of RNA and protein profiles and the general function of the protein(s). While this question is difficult to address globally, it can be approached by determining whether concordant RNA/protein pairs are found to be more frequently associated with specific gene ontology (GO) biological process terms. To address this, we first evaluated the overall coherence for the RNA and protein expression profiles separately within each biological process category using a logistic regression model. Table 1 shows that for only one biological process, “transcription”, the gene expression data for all 23 genes within the process were significantly fit to a single logistic regression model. However, the LC-FTICR data show good fit for protein abundance profiles within the “primary metabolism”, “protein metabolism” and “stress response” processes. Next, we determined the degree to which particular biological processes showed positive concordance using a Wilcoxon rank sum test between concordance score (F-statistic) and category membership. For these comparisons, the frequency with which each functional category was represented in the overall dataset was used to normalize the results.

**Table 1:** Summary of statistical analysis of concordance patterns among GO biological process categories.

Biological Process	# of RNA/protein pairs	MicroarrayLR	FTICRLR	ProcessConcordance
Cell adhesion/structure	52			
Intracellular transport	21			
Primary metabolism	22		XX	−
Protein metabolism	47		XX	+
Signal transduction	36			
Stress response	17		XX	
Transcription	23	X		
Unclassified	17			

LR: logistic regression; X: p<0.05; XX: p<0.01.

+: more concordance than expected by chance.

−: less concordance than expected by chance.

RNA/protein pairs in the biological process “protein metabolism” displayed more positive concordance than predicted by chance. Examples include proteins involved in protein processing and translational regulation of protein synthesis, such as eukaryotic translation initiation factor 4E (EIF4E) and ribosomal binding protein 1 homolog (RRBP1). In contrast, RNA/protein pairs in the “primary metabolism” category showed more anti-correlated patterns of expression than predicted by chance. These included proteins involved in fatty acid and carbohydrate metabolism, such as acetyl-coenzyme A acetyltransferase 1 (ACAT1) and pyruvate dehydrogenase alpha 1 (PDHA1). RNA/protein pairs whose annotation involved “signal transduction”, “stress response”, or “intracellular transport” processes, however, showed no statistically significant trend toward either concordant or discordant abundance changes based on the Wilcoxon rank sum test. These results provide strong support for previous assertions that the concordance between RNA and protein expression varies between specific functional classes of proteins [33], [34]. However our results are in contrast to those obtained in yeast, where genes associated with protein synthesis tend to show discordance between mRNA and protein abundance [27].

### Pathway and Network Analysis

**Figure 5:**
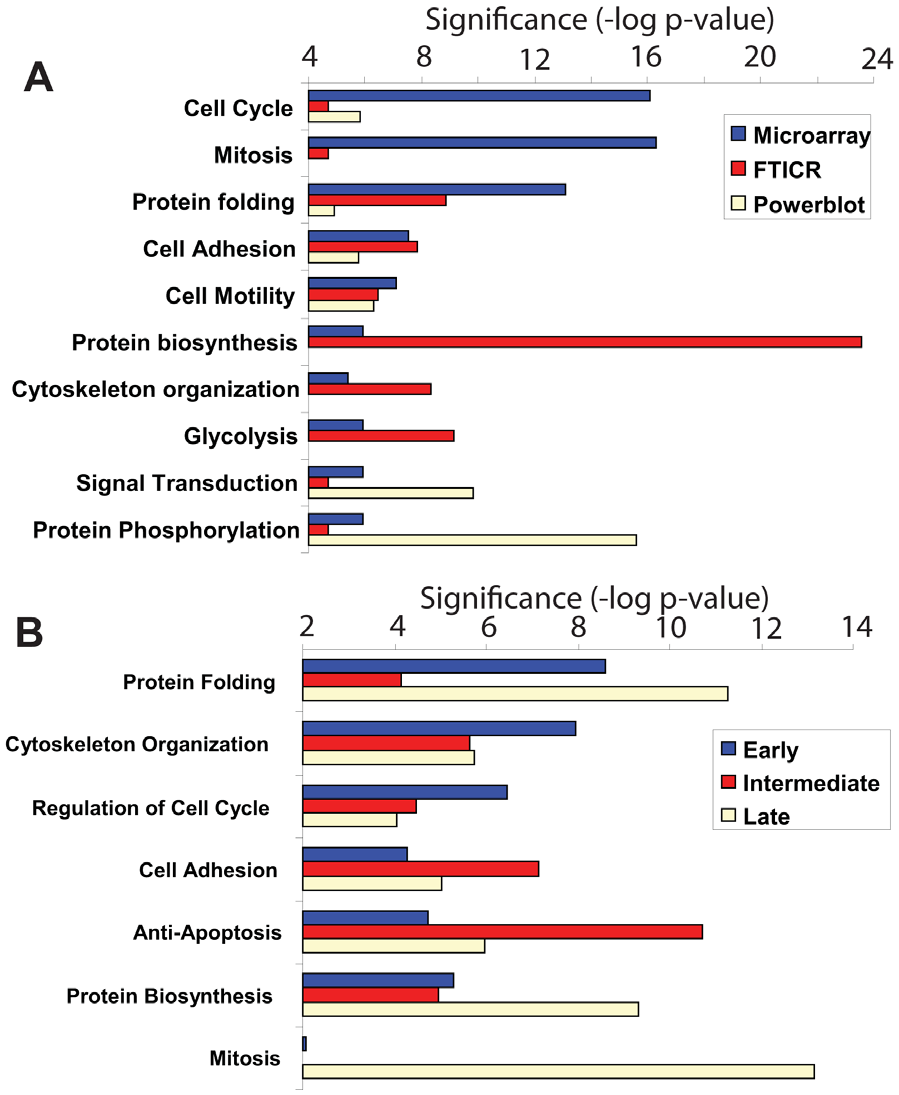
Major cell processes represented by each high-dimensional dataset. The biological processes represented by each data type across all time points (Panel A) were determined by gene set enrichment and significance values are p-values calculated within the MetaCore software. Only the cell processes showing the highest significance values are shown. The results in panel B show the major cell processes for all combined data, separated based on early (0–4 hr), intermediate (8–13 hr) or late (18–24 hr) time points after EGFR activation.

To determine the extent to which we could reconstruct the known EGFR regulatory networks from our datasets, we used the MetaCore software suite (GeneGo, St. Joseph, MI), which uses a curated, literature-based database of interaction and regulatory relationships to generate network maps from sets of differentially expressed genes or proteins. This approach was necessary because the limited number of conditions represented by the proteomic and microarray data in this study is insufficient to computationally infer connectivity between gene expression and protein expression nodes. We first evaluated whether similar biological processes regulated by EGFR activation are captured by each high-dimensional data platform by conducting gene set enrichment analysis on each dataset separately. The results (summarized across all time points) demonstrate that each data type emphasizes a specific pattern of cellular processes (Fig. 5A). The most significant biological processes in the microarray data include “cell cycle”, “mitosis” and “protein folding”, which were poorly represented by both proteomic datasets. The biological process most significant from the LC-FTICR data was “protein biosynthesis”, which in turn was poorly represented by the microarray and antibody-based proteomic results. The most significant processes from the antibody-based analysis were “signal transduction” and “protein phosphorylation”, and neither process was highly represented by either microarray or LC-FTICR analysis. Cellular processes associated with cell adhesion and cell motility were similarly represented by data across all measurement platforms.

To determine whether the pathway analyses of our integrated datasets made biological sense given our current understanding of EGFR signaling, we also analyzed how the major cell processes evolved over time. For these analyses, the major cell processes represented by the combined data were statistically ranked according to early (0–4 hr), intermediate (8–13 hr) and late (18–24 hr) time domains after EGFR activation (Fig. 5B). Significantly, the combined results recapitulate many of the expected cell processes associated with mitogenic and motility responses regulated by EGFR activation in these cells [20], [21], [22]. For example, “cytoskeletal organization” and “protein folding” (which includes protein chaperones involved in signal transduction) processes are highly represented soon after EGFR stimulation. Interestingly, anti-apoptotic pathways are significantly increased at times preceding entry into S-phase. Further, the most highly represented biological process in the 18–24 hr time domain is “mitosis”, consistent with the flow cytometry analysis results.

The results in Fig. 5 clearly demonstrate that different types of high-dimensional data provide qualitatively different views of the cell processes regulated by EGFR. However, to determine whether combined datasets can provide a more integrated understanding of EGFR signaling networks, we needed to first analyze the network structures obtained from each dataset individually. We evaluated how the connectivity of the inferred signaling networks differed among the individual datasets, and then how this changed when the datasets were merged. This overall connectivity reflects the ability of the data to accurately and comprehensively reconstruct the cell response networks. For this analysis, the individual and combined results from the early time domain (0–4 hr) data were used to infer regulatory networks based upon direct regulatory interactions between “nodes” (regulatory molecules) and “edges” (interactions) within the MetaCore database, however similar results were obtained for the middle and late time domains.

**Figure 6:**
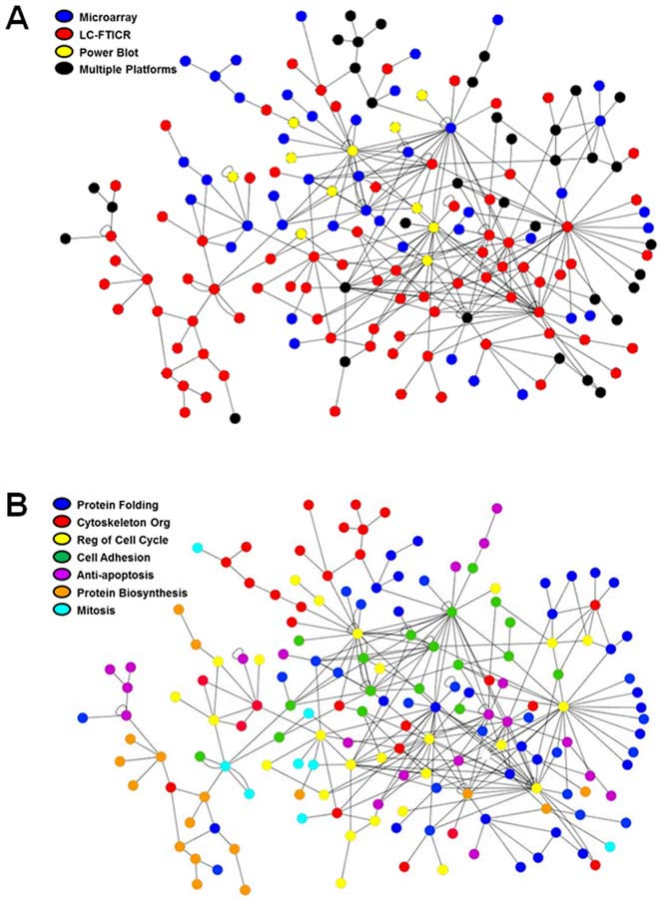
Example network inferred from the integrated datasets. Shown is the overall structure of one of the largest network clusters identified from the combined microarray, LC-FTICR and Powerblot datasets from the 0–4 hr early time domain. A) The source of the data contributing to each element (node) in this network cluster is coded by color, and connections between elements (edges) were inferred from the literature using the MetaCore database. B) The cellular processes represented by each node in the network are coded by color, using the process categories from Figure 5.

An example of the general network structure and connectivity of the major networks derived from early time domain data is shown in Fig. 6. The results in Table 2 summarize the network statistics obtained from this analysis. The largest network derived from the microarray data alone was a cluster of 50 nodes, out of 311 nodes with identified edges. The LC-FTICR data produced a smaller cluster of 19 nodes out of a total of 185 nodes with edges, consistent with the smaller number of overall changes identified by LC-FTICR as compared to microarray. The most highly linked nodes (hubs) in the microarray data were transcription factors, including FOS (15 edges) and EGR1 (10 edges). In contrast, the largest LC-FTICR network cluster included membrane-bound proteins, including EGFR (7 edges) and alpha-6, beta-1 integrin (7 edges) as the hub nodes. Thus, in the absence of the proteomics data, microarray data alone did not call out EGFR as a signaling node in the mitogenic response to EGF. Combining these two datasets generated a large cluster of 142 nodes, with the connectivity of the highly linked *EGFR* node from the LC-FTICR data increasing dramatically to 16 edges. When the microarray, LC-FTICR and PowerBlot data were all combined, the largest network cluster grew only modestly to 169 nodes. However, the connectivity of the highly linked nodes increased to 21 edges for EGFR and 28 edges for FOS (Table 2).

**Table 2:** Summary of network statistics derived from individual and integrated datasets.

	Microarray alone	LC-FTICR alone	Microarray+LC-FTICR	Microarray+LC-FTICR+PowerBlot
Total number of nodes	311	185	476	494
Size of largest cluster	50	19	142	169
% of nodes in largest cluster	16	10	30	34
Number of edges	156	53	432	575
Degree of largest cluster	3.12	2.79	3.04	3.40
Two Primary hub nodes (# of edges)	*FOS* (15) *EGR1* (10)	*EGFR* (7) *ITGB1* (7)	*FOS* (18) *EGFR* (16)	*FOS* (28) *SRC* (25)

One of the most highly linked nodes in the largest network cluster was SRC (25 edges), which emerged from the protein phosphorylation data obtained from the antibody analysis (Fig. 6). Other well-connected hub nodes represented by phosphorylation data alone included STAT3 and ERK2. Merging the microarray, LC-FTICR and PowerBlot data also increased the overall connectivity of the networks, with 34% of the nodes included in the largest network cluster compared to only 16% with microarray alone (Table 2). In addition, the degree of the largest network clusters increased to an average of 3.4 edges per node.

Each data type also contributed to different types of edges in the overall inferred network. For example, from the microarray data alone, 63% of the edges were inferred to be transcriptional regulatory relationships whereas only 17% were inferred to be direct protein interaction events. In contrast, 60% of the edges from the LC-FTICR data were inferred to represent protein interaction events and only 4% to represent direct transcriptional regulation. These findings are not unanticipated, but they provide an indicator of the added value of each data type in the reconstruction of cell response networks.

Since the primary goal of our study was to quantify the benefits of data integration across different measurement platforms for network and pathway analysis, the EGFR system was a valuable system due to the extensive knowledge of signaling networks it is coupled to. However, a surprising observation from our combined results was that the most robust response to mitogenic concentrations of EGF was not cell cycle regulation, but induction of matrix metalloproteinase cascades. Indeed, RNA levels for interstitial collagenase (MMP1), and stromelysin 2 (MMP10) were among the largest changes observed in the study, increasing 24- and 8-fold, respectively. To validate these high-throughput measurements, additional experiments were conducted using orthogonal techniques to measure the secretion of MMP1 and MMP10 protein. ELISA analysis of conditioned medium collected from HMEC during mitogenic stimulation confirmed that high levels of both MMP1 and MMP10 protein are secreted in a manner that is dependent on EGFR kinase activity (Figure S2).

## Discussion

The concept that integration of data derived from multiple levels of biological regulation will improve our understanding of signaling networks is generally accepted, yet is rarely practiced in biological research. To our knowledge, this study is the first systematic analysis of the practical benefits of merging heterogeneous temporal data for purposes of network and pathway interrogation in human cells. We focused on the EGFR pathway because it plays an important role in epithelial cell regulation and cancer biology, and there is mounting evidence that EGFR transactivation is coupled to wide variety of external stimuli [20], [35]. The extensive literature on this pathway also provided a means for validating our pathway analysis results. We demonstrate that data from different types of high-dimensional platforms independently provided qualitatively different views of EGFR-induced cell processes and pathways. However, when data representing RNA regulation, protein abundance and protein phosphorylation were combined, the results recapitulate the major processes and signaling networks known to be regulated by EGFR in this cell type.

An important reason for integrated data analysis is that RNA abundance changes are not always a good predictor of protein abundance changes, especially over a time scale of several hours. The canonical correlation analysis we describe here has advantages over simple correlation analysis, since it can conceptually capture RNA and protein expression profiles that are “concordant” yet out of sync due to temporal delays. The canonical correlation of 0.44 found in this study is greater than correlations previously reported in which the slopes of RNA and protein temporal profiles were used for comparison [33]. However, even when temporal shifts between RNA and protein expression profiles were considered for the EGF-stimulated state through statistical modeling of time course data, we found that no more than half of the protein abundance changes measured by LC-FTICR were accompanied by corresponding RNA changes. These collective results suggest that a high degree of posttranscriptional regulation is involved in the response of mammalian cells to EGF.

Beyond general correlations, our results also suggest that some functional classes of proteins show a greater tendency toward tight coupling to transcriptional control than others. Most studies reporting global measurements of gene and protein expression levels have used single time point designs and rely on steady-state measurements that do not discriminate between synthesis and degradation processes that underlie changes in abundance (for review see Waters et al, 2006). However, rates of protein degradation and turnover can vary by orders of magnitude, even with highly abundant proteins [36]. It was previously proposed that for cell structural proteins there is a greater correlation between protein and RNA expression changes than was predicted from overall RNA/protein expression profiles [33], [34]. Our analyses focused on a subset of 199 RNA/protein pairs where complete time course data was obtained, to avoid compounding errors associated with extrapolation across missing data. This subset of data is expected to be generally enriched with higher abundance proteins. Proteins whose GO annotation included cell structure and cell adhesion (52 total) were indeed the most highly represented in this group, and included proteins such as tubulin and actin subunits, and moesin. Still, when the frequency of detection (i.e., spectra counts) was used as a means for normalizing for abundance, we found no statistically significant trend toward either concordant or discordant patterns of RNA and protein temporal changes for these functional classes of molecules. In contrast, RNA/protein pairs that showed more concordant temporal profiles were statistically enriched with proteins involved in protein synthesis and processing. Furthermore, RNA/protein pairs that showed anti-correlated expression patterns were also observed, and included a greater fraction of proteins involved in primary metabolic processes associated with fatty acid and energy metabolism than expected by chance. As a functional class, primary metabolism proteins were not disproportionably represented in the subset of RNA/protein pairs evaluated (22 total). Thus, these results are not readily explained by ‘sampling frequency’ or by a general increase in mass spectrometry measurement accuracy for abundant proteins. Admittedly, inaccuracies and generalizations of GO annotation terms limit their use for defining functional classifications. Nonetheless, the results support the hypothesis that specific cell processes may differentially utilize transcriptional and post-transcriptional mechanisms of control.

The major cell processes found to be modulated by EGFR were qualitatively very different with respect to each measurement platform (Fig. 5A). The emphases on different cell processes by each platform are in part attributed to different depths of coverage across the genome or proteome. As expected, microarray analysis identified over 3- and 15-fold greater numbers of gene products that changed compared to MS or Western blot analysis, respectively. While some cell processes, such as cell adhesion and cell motility, were similarly represented in all datasets, other processes were preferentially represented by a specific dataset. For example, less abundant and transiently induced proteins typical of cell cycle regulation were not readily identified by either LC-FTICR or antibody-based analysis, whereas microarray analysis identified a large number of gene products associated with this process. A strong bias toward signal transduction processes was also highly apparent in the Western blot data (Fig. 5A), reflecting a more general bias of commercially produced antibodies toward signal transduction proteins. Despite the biases introduced by individual datasets, the composite pathway analysis results included most of the expected cell processes induced by EGFR in this model system. For example, the combined results (Fig. 5B) illustrate that following EGFR stimulation there is a general shift in the major cell processes from cytoskeletal organization and cell cycle regulatory processes during initial times (0–4 hr) toward anti-apoptotic and cell adhesion pathways (8–13 hr). The temporal evolution of these processes is consistent with the initial entry of cells into S-phase, which begins at ∼13 hr. Similarly, the increased representation of mitosis pathways between 18–24 hr corresponded to an increase in mitotic cells as monitored by flow cytometry.

An important advantage of data integration observed here is that the networks derived from heterogeneous datasets show a more connected topology than networks derived from a single dataset. Cellular signaling networks are thought to approximate a scale-free topology, characterized by fewer nodes with a higher degree of connectivity [37]. In this respect, networks inferred from microarray data alone have the advantage of greater genomic coverage compared to data from even the most advanced proteomic platforms. Although networks derived from LC-FTICR or antibody data alone had fewer nodes and less connectivity, these datasets contributed important qualitative features to the overall network structure, mostly because of their ability to indicate the activity state of different signaling networks. Network analysis of the integrated data revealed SRC and STAT3 as among the most linked signaling nodes, consistent with previous studies in HMEC [10]. It is noteworthy that these signaling nodes were determined primarily from a subset of the Western blot results that used phospho-specific antibodies. Despite the limited overall amount of protein phosphorylation data, the topology of the signaling networks derived from the integrated results was highly dependent on these data. Other recent studies have also reported novel findings through the incorporation of phosphorylation data into their analysis of disease and signaling networks [38], [39]. Given that dynamic modification of cellular proteins with phosphate is one of the key regulators of cellular response, it is not surprising that signaling network reconstruction is highly dependent upon the collection of these data.

In contrast to the microarray results, 60% of the node-edge relationships identified from the LC-FTICR data infer direct protein interactions as regulatory mechanisms. The bias of LC-FTICR results towards protein interactions is further implied from network statistics showing that there were nearly 3-fold more nodes with multiple edges (multiple effectors) than nodes with single edges (binary interactions). This also contrasts the microarray data, where the fraction of inferred nodes with multiple edges was only 1.6-fold greater than nodes with single edges. It is noteworthy that based on microarray data alone, EGFR would not have been identified as a major node in the pathway analysis. However, the largest network clusters identified from the LC-FTICR data alone included EGFR itself and β1-integrin as the most connected nodes. Previous studies have reported that EGFR and β1-integrin associate as a complex, providing a mechanism for bidirectional coupling of EGFR signaling to the extracellular matrix and other receptor pathways [40], [41]. Activation of EGFR by integrin complexes is thought to require *SRC* as a signaling intermediate [40], [41], consistent with our integrated network analysis, which implicates SRC as a central node in the HMEC EGFR network. We also found that the most robust responses associated with EGF-induced mitogenesis were not classical cell cycle regulators, but metalloproteases capable of remodeling the local extracellular environment. This is in contrast to previous microarray-based studies examining EGF responses of transformed cells that found that induction of negative feedback inhibitors of EGFR was a dominant response [42]. However, unlike HMEC, cells in that previous study do not respond mitogenically to EGF. The strong coupling of MMP cascades to the mitogenic response observed in our study may be particularly important for understanding the relationships between amplified EGFR signaling and invasiveness of many epithelial cancers.

A limitation of pathway analysis using computational tools based on literature-curated databases is that relationships involving non-annotated molecules cannot be identified. However, such tools are highly useful for weighting abundance changes for specific gene products identified experimentally and inferring signaling linkages that are not always intuitive. By using curated databases for comparison, our study illustrates some of the strengths and biases inherent in different measurement platforms. Despite platform-specific strengths and weaknesses, integration of heterogeneous datasets not only enhances the connectivity of the derived networks, but also qualitatively influences the regulatory relationships inferred from high-dimensional data. Computational inference of signaling networks would be a preferable strategy to utilize all of the data, however inference utilizing multiple data types is challenging due to differences in quantitative range and sheer volume of data required for robust calculations [19], [43], [44], [45]. Despite the limitations of our study, the results underscore the importance of data integration across multiple molecular levels for reconstructing signaling networks that describe cellular behavior. Future studies will focus on the use of dynamical data (multiple time points and conditions) and computational inference of disease networks from integrated data.

## Materials and Methods

### Materials and Cell Treatment

The human mammary epithelial cell line HMEC (strain 184A1), obtained from Martha Stampfer (Lawrence Berkeley National Laboratory), [46] was used in this study. These cells are non-tumorigenic and are dependent on EGFR signaling for normal proliferation and motility in culture [20], [21], [22]. Cells were routinely cultured in DHFB-I medium supplemented with 12.5 ng/ml EGF as described [20]. All other reagents were of cell culture grade or higher quality.

For the experiments shown, 7.5×10^4^ cells were seeded in 150-mm dishes and allowed 24 hr in complete medium for attachment and growth. Growth arrest was induced by replacing the medium with medium lacking serum, EGF and other growth factors for 48 hr. A mitogenic response was then initiated by treating cells with 10 ng/ml EGF in minimal medium. Samples were collected at 0, 0.25, 1, 4, 8, 13, 18 and 24 hr and processed for RNA expression profiling and proteomic analyses as described below. The fraction of cells in G_0_/G_1_, S and G_2_/M phases of the cell cycle was measured in parallel samples by flow cytometry analysis (Becton Dickinson FACSCalibur) after fixation in cold 70% ethanol and labeling with propidium iodide.

### Microarray Analysis

Total RNA was prepared using a Qiagen RNeasy Mini kit (Qiagen, Valencia, CA, USA) and the integrity and purity evaluated by gel electrophoresis and absorption spectroscopy. RNA expression profiles were analyzed using NimbleGen whole genome 60-mer oligonucleotide arrays (Design Version 2003_10_27) which contains 38,108 array elements (NimbleGen, Madison, WI). Briefly, 0.5 µg of total RNA for each sample was used for cRNA synthesis using oligo-dT primers and T7 RNA polymerase through the NimbleGen Microarray Service facility. Each biological sample was hybridized against 3 independent arrays (3 technical replicates), and quality control assessment determined that the arrays for the 15 minute time point failed to hybridize according to manufacturer specifications. Raw intensity data for the remaining arrays were quantile normalized [47] and subjected to pairwise analysis of variance with a 5% false discovery rate calculation [48] to identify significantly changed genes.

### Western Blot Analyses

For analysis of protein abundance changes, cell pellets collected at different time points were separately washed three times with ice-cold phosphate-buffered saline. The cells were lysed in buffer (10 mM Tris, 150 mM NaCl, 1% NP-40, 1 mM NaVO_3_, 10 mM NaF, and protease inhibitor cocktail, pH 7.4) using intermittent sonication on ice. The lysates were centrifuged for 20 min at 4°C at 14000 g to pellet any residual debris. Protein concentrations were determined using the BCA assay (Pierce Biochemical). Parallel Western blot analyses were performed by BD Biosciences (San Diego, CA) through their PowerBlot™ custom service using the complete array of available antibodies (1011 total). This approach employs co-hybridization of panels of antibodies that recognize distinctly migrating proteins on denaturing gradient gels, followed by detection with secondary antibody and quantification by gel image analysis. Samples were analyzed in triplicate, and gel image analysis of expression ratios were determined using a 3×3 matrix comparison method to determine the ratio of treated∶control (0 hr) for each of the triplicate analyses. Only proteins which passed visual inspection of the image quality and showed a ≥1.5-fold change compared to control (0 hr) were considered significant. The raw microarray data files are publicly available in GEO (GSE15668), and significant gene lists used in the bioinformatics analyses are included as Table S1 with this manuscript.

### Mass spectrometry-based proteomic analysis

For MS-based proteome analysis, we used the accurate mass and time (AMT) tag approach [23]. The approach uses the high mass measurement accuracy of Fourier-transform ion cyclotron resonance-mass spectrometry (FTICR-MS), coupled with advanced capillary liquid chromatography (LC) separations to identify and estimate the abundance of peptides. An existing AMT tag database encompassing the monoisotopic mass and normalized chromatographic elution times of peptides identified from previous LC-MS/MS analyses of HMEC proteins under a range of experimental conditions [9], [24], [25], [26] was used as a base reference database for the LC-FTICR measurements in this study. The existing HMEC database was further enriched by conducting additional LC-MS/MS analysis using 375 µg of HMEC protein pooled from each of time point samples in this study. Details of LC-MS/MS analysis and data filtering involved in peptide identification have been described elsewhere [26]. Peptide identifications in this database were obtained using the SEQUEST algorithm to independently search all MS/MS spectra against the human International Protein Index (IPI) database and against the reversed-sequence human IPI protein database to estimate the false discovery rate. Criteria that would yield an overall confidence of over 99% were established for filtering raw peptide identifications at spectra level. The peptide retention times from each LC-MS/MS analysis were normalized to a range of 0–1 using a predictive peptide LC-normalized elution time (NET) model and linear regression as previously reported [49].

For quantitative analysis by LC-FTICR, proteins from each of the eight lysates were digested with trypsin separately and cysteinyl and non-cysteinyl peptides was prepared from the tryptic digests and were differentially labeled separately using post-digestion trypsin-catalyzed ^16^O-to-^18^O exchange as previously described [9]. The control sample (0 hr) was labeled with ^16^O and all the other samples (t = 0.25, 1, 4, 8, 13, 18 and 24 hr) were labeled with ^18^O. Supernatants were collected by centrifuging the samples for 5 min at 15000 g, and equal amounts of the ^16^O-labeled control sample were combined with each of the ^18^O-labeled time point samples, and dried.

Samples were analyzed using an in-house 11.5-T FTICR mass spectrometer. The same LC system and gradient was applied for LC-MS/MS and LC-FTICR analysis. The LC-FTICR data analysis was conducted as previously described [50]. Briefly, the initial analysis of raw LC-FTICR data involved a mass transformation or deisotoping step using in-house software (ICR2LS). The ICR2LS analysis generates a text file report for each LC-FTICR dataset that includes the monoisotopic masses and the corresponding intensities for all detected species for each spectrum. In-house software (VIPER) was used to detect LC-MS features (i.e., a peak with unique mass and elution times) and assign them to peptides in the AMT database. Data processing steps included filtering data based on isotopic fitting, finding features and pairs of features, computing abundance ratios for pairs of features (^16^O∶^18^O), normalizing LC elution times, and matching the accurate measured masses (±5 ppm) and NET (±2%) values of each feature to the corresponding mass and time tag in the database to identify peptide sequences. All identified peptides were assigned an identical probability of 1.0 and entered into ProteinProphet software [51] to remove redundant proteins. Protein abundance ratios were calculated as an average of the isotopic ratios of both the cysteinyl and non-cysteinyl peptides after removing outliers using Grubb's test. For final network and pathway analysis, only proteins identified by at least two peptides and those that showed a ≥1.5-fold change compared to control (0 hr) were used. The proteomic data generated in this study are publicly available at http://omics.pnl.gov, and final datasets used in the bioinformatics analyses are submitted as Table S1 with this manuscript.

### Bioinformatic and statistical analysis

Data were integrated across platforms using the Bioinformatics Resource Manager software [52]. Hierarchical and Kmeans cluster analyses of microarray and proteomic data were performed using OmniViz (Maynard, MA) and Multi-Experiment Viewer [53] software and were based on log_10_ expression ratio values. Biological process enrichment statistics were calculated using the pathway mapping tool, MetaCore (GeneGo, St Joseph, MI) based upon a hypergeometric distribution where the p-value represents the probability of particular mapping arising by chance, given the number of significantly regulated genes/proteins in the dataset, the total number of genes assigned to a process, and the background genes/proteins experimentally identified by the platform. Networks were reconstructed using MetaCore's direct interaction algorithm, where the only edges allowed are those between two root nodes (e.g., objects from the list directly connected to each other) using their proprietary database of curated interactions from the literature. The resulting integrated network was redrawn using Cytoscape [54] for illustration.

Canonical correlation analyses of time profile microarray and proteomic data were conducted using the freely available R software (version 1.7.1; R Development Core Team, 2004). Prior to analysis all time profiles were individually transformed to have mean 0 and standard deviation 1, so that subsequent comparisons emphasize the shapes of the profiles. Canonical correlation analysis (using the *cancor* function in R) was used to assess the overall (global) concordance of RNA and protein temporal expression profiles. The significance of the observed canonical correlations was evaluated by permuting protein labels (but keeping time profiles intact), and computing the canonical correlations for each of 1000 permutations. Genes were ranked according to the concordance of their individual RNA and protein expression profiles. Rankings were made by reference to a smoothing model. Let 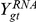 and 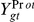 denote the scaled 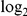 expression values at time *t* for gene *g* for RNA and protein, respectively. The respective time profiles were fit to the models (using the *lm* function in R)
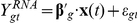


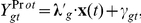
where 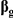 and 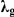 are parameter vectors, estimated from the data, describing the shapes of the corresponding RNA and protein time profiles, and the regression vector 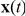 is a spline basis function with 3 degrees of freedom. Two criteria were used to rank genes. The first is whether 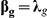, that is, that the two smoothed profiles have identical shape. The second is the overall significance of the regression (across both RNA and protein profiles) given that 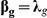. Rankings were based on an F-test statistic reflecting the tradeoff, or compromise, between the two criteria. We constructed a (data-determined) composite measure as a scalar function of F1 and F2 to rank genes according to RNA-protein concordance. This new measure is of the form 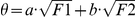 where *a>0* and *b<0*.

Enrichment of protein functional groups was then assessed for RNA and protein time profiles as a function of their concordance, relating the F1 score of concordance to gene ontology category. For each gene we computed two F1 scores: one for positive concordance (given in Table 1) and one for negative concordance. These scores are related separately to GO category and are both given for the example gene/protein pairs in Figure S1. For positive concordance within each process, the relationship between concordance score F1 and category membership (0 or 1) was assessed separately for each category using the Wilcoxon rank sum test.

## Supporting Information

Figure S1 **Example RNA and protein expression profiles showing correlated (A) and anti-correlated (B) temporal patterns.** The x-axis is time in hours. The left-hand y-axis is the RNA scale, and the right-hand y-axis is the protein abundance scale. The RNA profile is shown in red and protein profile is in blue. The dashed line indicates the regression-based fit for temporal concordance. Values are expressed as log_2_ expression ratios.
(TIF)

Figure S2 **EGFR-Regulated Secretion of Matrix Metalloproteases.** HMEC were treated with EGF (10 ng/ml), alone or in the presence of the selective EGFR kinase inhibitor PD153035 (200 nm). MMP-1 and MMP-10 protein levels were measured in conditioned medium at 24 hr using ELISA (R&D Systems, Minneapolis, MN). Values are the mean ± s.d. of biological triplicates.
(TIF)

Table S1 **Significant gene and protein lists measured by microarrays, FTICR proteomics and western blot analysis in the time course study.** The file includes gene/protein identifiers from each platform and values listed are log_10_ expression ratios relative to time 0 hr controls.
(XLS)
