# Supplementary figures and images for: Network Analysis of Epidermal Growth Factor Signaling Using Integrated Genomic, Proteomic and Phosphorylation Data
Source: PLoS One. 2012 Mar 29;7(3):e34515. doi: 10.1371/journal.pone.0034515 (PMC3315547; doi:10.1371/journal.pone.0034515)

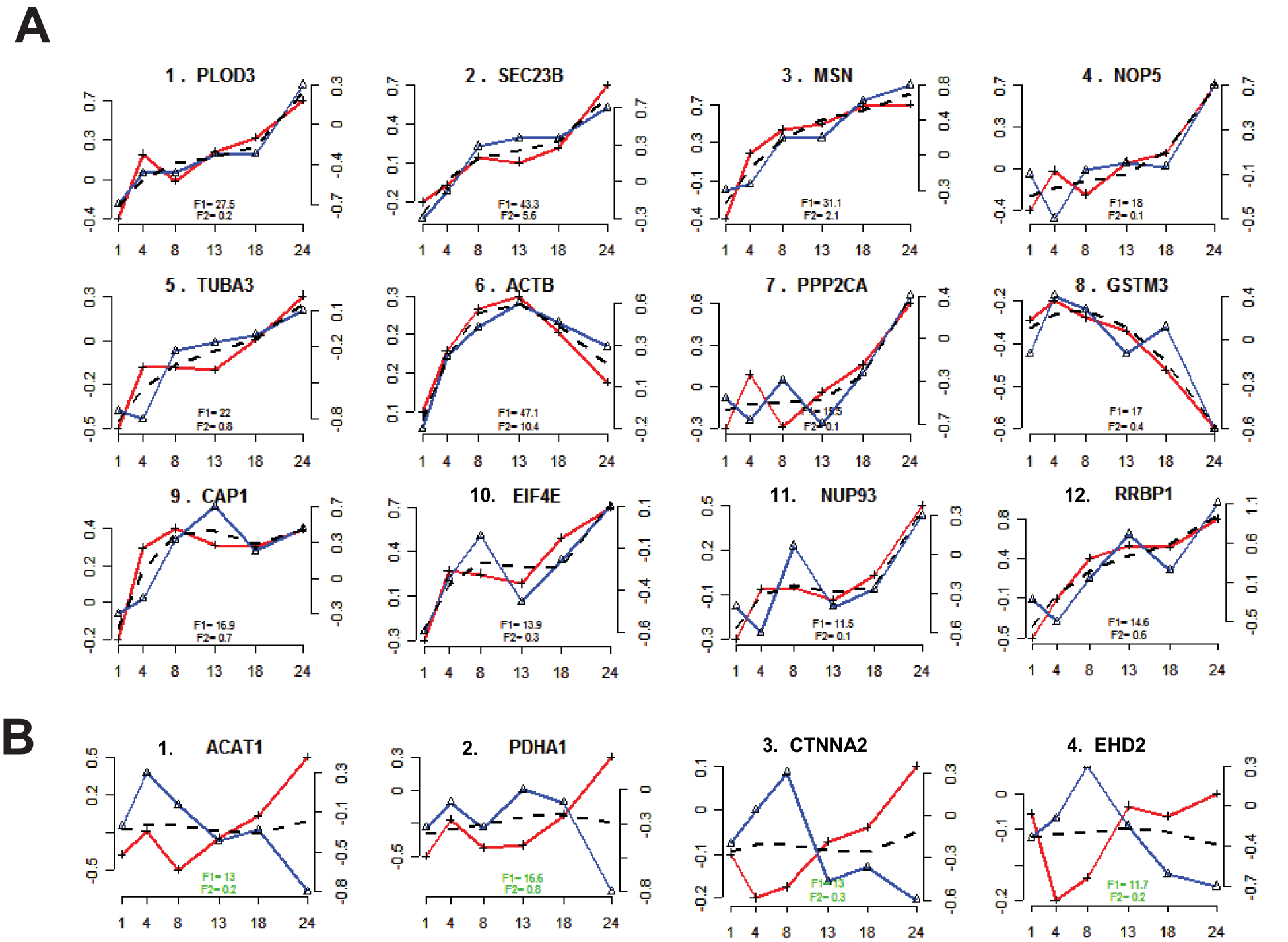

Supplement: Figure S1 — Example RNA and protein expression profiles showing correlated (A) and anti-correlated (B) temporal patterns. The x-axis is time in hours. The left-hand y-axis is the RNA scale, and the right-hand y-axis is the protein abundance scale. The RNA profile is shown in red and protein profile is in blue. The dashed line indicates the regression-based fit for temporal concordance. Values are expressed as log2 expression ratios. (TIF) [file pone.0034515.s001.tif]

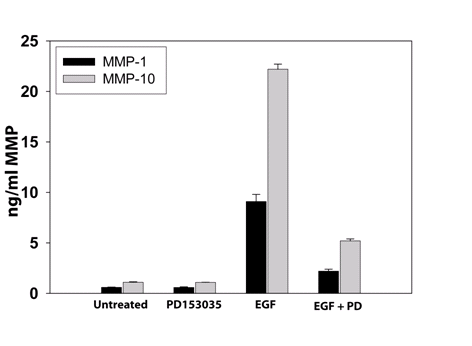

Supplement: Figure S2 — EGFR-Regulated Secretion of Matrix Metalloproteases. HMEC were treated with EGF (10 ng/ml), alone or in the presence of the selective EGFR kinase inhibitor PD153035 (200 nm). MMP-1 and MMP-10 protein levels were measured in conditioned medium at 24 hr using ELISA (R&D Systems, Minneapolis, MN). Values are the mean ± s.d. of biological triplicates. (TIF) [file pone.0034515.s002.tif]
